# Understanding Health Care Disparities Based on Medicare Use for Inflammatory and Infectious Eye Diseases

**DOI:** 10.1167/tvst.13.8.34

**Published:** 2024-08-22

**Authors:** Krati Chauhan, James T. Rosenbaum

**Affiliations:** 1 The University of Vermont-Larner College of Medicine, Burlington, Vermont, USA; 2 Legacy Devers Eye Institute, Portland, Oregon, USA

**Keywords:** inflammatory eye diseases, infectious eye diseases, health disparities, medicare, data science

## Abstract

**Purpose:**

Inflammatory and infectious eye diseases are an important cause of visual impairment in patients older than 65 years of age. Health care disparities for eye care are present for general eye care. However, there is lack of national data on health disparities regarding eye care use for inflammatory and infectious eye diseases. Our study examines the effect of gender and race on eye care in patients with inflammatory and infectious eye diseases who are equal or greater than 65 years of age.

**Methods:**

We have used Medicare data to examine the effect gender and race on use of eye care services in patients with inflammatory and infectious eye diseases for 2014 to 2018. Medicare is a national insurance program administered by the government of United States to insure people age 65 years or older. Owing to its high enrollment, those in Medicare are representative of the U.S. population aged 65 and older.

**Results:**

We found that females have higher use for Medicare for inflammatory and infectious eye diseases across all races from 2014 to 2018. On examining the effect of race, African Americans have lower use as compared with Whites. People of Asian descent have the highest use, followed by Hispanic people.

**Conclusions:**

Health care disparities exist for eye care use for inflammatory and infectious eye diseases for patients 65 years of age and older. Future studies are required to address these disparities to provide equitable eye care.

**Translational Relevance:**

Identification of eye care disparities is the first step to addressing these disparities.

## Introduction

Inflammatory and infectious eye diseases include conditions such as uveitis, retinitis, conjunctivitis, keratitis and orbital inflammation^1^ and can result in vision loss and disability. These conditions disproportionately affect older adults,^2^^,^^3^ and are an important cause of visual impairment in people who are 65 years of age and older.^4^

Previous studies have recognized racial and gender disparities in health care use for eye conditions like glaucoma. Several of these studies have been confined to a specific geographic region or to outpatient setting.^5^^–^^7^ There is limited research on the effects of race and gender on health care use for inflammatory and infectious eye diseases specifically focused on individuals greater than 65 years of age at a national level.

Determining the influence of race and gender on use for inflammatory and infectious eye diseases is crucial for identifying health care disparities. Examining health care use among different racial and gender groups will provide an understanding into the specific needs faced by these populations. This knowledge can be used to develop interventions for providing equitable eye care with the ultimate aim of improving visual outcomes for individuals’ 65 years of age and older affected by these sight threatening conditions.

The National Vision and Eye Health Surveillance System (VEHSS) provides a unique opportunity to examine these disparities. Our study aims to explore the effects of race and gender on Medicare use for inflammatory and infectious eye diseases using data from the VEHSS.

## Methods

We have used Medicare data available through the National VEHSS. Medicare is a national insurance program administered by the federal government in the United States to insure people primarily age 65 or older. In 2015, approximately 42.5 million of 47.8 million Americans aged 65 and older (88.9%) were enrolled in Medicare. Because of this high coverage rate, those enrolled in Medicare are representative of the overall population aged 65 and older in the United States. Routine eye examinations and optometry services are not covered by Medicare.

Medicare data were collected from research identifiable files obtained through the Centers for Medicare and Medicaid Services Virtual Research Data Center and include all fee-for-service beneficiaries. Fee for service is a system of health care payment in which a provider is paid separately for each specific service rendered. Patients must have been enrolled in the Medicare program for a full 12 months to be included in the study. To ensure patient privacy and protections, the Centers for Medicare and Medicaid Services requires suppression of denominators of less than 11 and these data have been suppressed for this study. Results were further suppressed for all the diagnoses if the numerator was 3 or less and the denominator was less than 30, or the numerator was 3 or greater and the denominator was 30 or less. For example, if there are three cases of orbital inflammation, reported in 2017, the data would be suppressed. If there are 5 cases of orbital inflammation reported in 25 cases of orbital disorders, the data would also be suppressed. This study adheres to the guidelines of the Declaration of Helsinki and is was done using deidentified, publicly available data from the VEHSS https://ddt-vehss.cdc.gov/LP?LocationId=59.

The VEHSS was established by a cooperative agreement with the Centers for Disease Control and Prevention and the Non-partisan and Objective Research Organization at the University of Chicago. The VEHSS uses the *International Classification of* *Diseases* (ICD), 9th and 10th edition, codes to identify ocular conditions. Diagnosis codes may be primary or secondary diagnoses.

These ICD codes are organized into two levels: category and subgroup. Each ICD code is assigned to one subgroup and multiple subgroups are combined to form a category. The inflammatory and infectious eye disease category includes subgroups of ocular inflammatory conditions (uveitis, scleritis, and episcleritis), keratitis, conjunctivitis, lacrimal system and orbital inflammation, eyelid inflammation and infection, infectious diseases, and endophthalmitis. Table 1 shows the ICD-9 and -10 codes assigned to each subgroup. Suppose a patient is diagnosed with keratitis and conjunctivitis for 2017. This patient is counted as one prevalent case in 2017 for the subgroups keratitis and conjunctivitis and one prevalent case for the category inflammatory and infectious eye disease. Hence, one patient may appear in multiple subgroups within one category, but cannot be double counted in one category. Other variables that are available include age, race, and gender.

**Table 1.** ICD-9 and ICD-10 Codes Included in Each Subgroup for the Category: Inflammatory and Infectious Eye Diseases

Subgroups	ICD-9 Codes	ICD-10 Codes
Keratitis	37000-37007, 37020-37024, 37031-37035, 37040, 37044, 37049, 37050, 37052, 37054, 37055, 37059, 37060-37064, 37080, 3709	H16001-H16003, H16009, H16041-H16043, H16049, H16021-H16023, H16029, H16011-H16013, H16019, H16031-H16033, H16039, H16061-H16063, H16069, H16071-H16073, H16079, H16051-H16053, H16059, H16101-H16103, H16109, H16141-H16143, H16149, H16111-H16113, H16119, H16121-H16123, H16129, H16131-H16133, H16139, H16251-H16253, H16259, H16221-H16223, H16229, H16211-H16213, H16219, H16231-H16233, H16239, H16201-H16203, H16209, H16291-H16293, H16299, H16301-H16303, H16309, H16321-H16323, H16329, H16331-H16333, H16339, H16311-H16313, H16319, H16391-H16393, H16399, H16401-H16403, H16409, H16431-H16433, H16439, H16421-H16423, H16429, H16441-H16443, H16449, H16411-H16413, H16419, H168-H169, H16, H160, H1600-H1607, H161, H1610-H1614, H162, H1620-H1626, H1629, H163, H1631-H1633, H1639, H164, H1640-H1644, H16261-H16263, H16269
Conjunctivitis	37200-37206, 37210-37215, 37220-37222, 37230-37234, 37239, 37240-37245, 37250, 37252, 37254, 37255, 37256, 37261-37264, 37271-37375, 37281, 37289, 37290	H1030-H1033, H10231-H10233, H10239, H10011-H10013, H10019, H10021-H10023, H10029, H10221-H10223, H10229, H1010-H1013, H10211-H10213, H10219, H10401-H10403, H10409, H10421-H10423, H10429, H10431-H10433, H10439, H1044, H10411-H10413, H10419, H1045, H1089, H10501-H10503, H10509, H10521-H10523, H10529, H10531-H10533, H10539, H109, H10811-H10813, H10819, H10511-H10513, H10519, H11001-H11003, H11009, H11011-H11013, H11019, H11041-H11043, H11049, H11051-H11053, H11059, H11021-H11023, H11029, H11031-H11033, H11039, H11061-H11063, H11069, H1110, H11159, H11811-H11813, H11819, H11141-H11143, H11149, H11121-H11123, H11129, H11131-H11133, H11139, H11111-H11113, H11119, H11221-H11223, H11229, H11211-H11213, H11219, H11231-H11233, H11239, H11241-H11243, H11249, H11431-H11433, H11439, H1130-H1133, H11421-H11423, H11429, H11411-H11413, H11419, H11441-H11443, H11449, H11821-H11823, H11829, H1189, H119, B0231, H10, H100, H1001, H1002, H101, H102, H1021-H1023, H103, H104, H1040-H1043, H105, H1050-H1053, H108, H1081, H11, H110, H1100, H1101-H1106, H111, H1112-H1115, H11151-H11153, H112, H1121-H1124, H113-H114, H1141-H1144, H118 H1181-H1182, P391
Other inflammatory conditions	36300-36301, 36303-36308, 36310-36315, 36320-36322, 36400-36405, 36410-36411, 36421-36424, 3643, 37900-37907, 37909	H30001-H30003, H30009, H30011-H30013, H30019, H30021-H30023, H30029, H30031-H30033, H30039, H30041-H30043, H30049, H30101-H30103, H30109, H30111-H30113, H30119, H30121-H30123, H30129, H3013-H30133, H30139, H30141-H30143, H30149, H30891-H30893, H30899, H3090-H3093, H3020-H3021, H3022-H3023, H30811-H30813, H30819, H2000, H20011-H20013, H20019, H20021-H20023, H20029, H20031-H20033, H20039, H20041-H20043, H20049, H20051-H20053, H20059, H2010-H2013, H209, H20811-H20813, H20819, H2020-H2023, H20821-H20823, H20829, H15001-H20053, H20059, H2010-H2013, H209, H20811-H20813, H20819, H2020-H2023, H20821-H20823, H20829, H15001-H15003, H15009, H15101-H15103, H15109, H15111-H15113, H15119, H15121-H15123, H15129, H15011-H15013, H15019, H15051-H15053, H15059, H15041-H15043, H15049, H15021-H15023, H15029, H15031-H15033, H15039, H15091-H15093, H15099, H01, H1500-H1505, H1509, H151, H1510-H1512, H20, H200, H2001-H2005, H201-H208, H2081-H2082, H30, H300, H3000-H3004, H301, H3010-H3014, H302, H308, H3081, H3089, H309, H4040 × 0
Lacrimal system and orbital inflammation	173, 532, 544, 76, 77, 779, 984, 190, 24901, 24911, 24921, 24941, 3601-3606, 3608, 3610-3611, 3613, 3618, 3620-3624, 3626-3627, 3630-3632, 3635-3637, 3640-3642, 3644-3656, 3658, 3660-3665, 3672-3673, 3675, 3678, 3680-3681, 3683-3686, 3690-3692, 3696, 3697, 370, 3700, 3702-3706, 371, 3711-3716, 372, 3720-3728, 373, 3730-3731, 3733, 374, 3740-3745, 3748, 3750, 37500-37503, 3751-3753, 37530-37533, 3754, 37541-37543, 3755-3756, 3758, 3760, 37600-37604, 3761, 37610-37612, 3762-3765, 3768, 3770-3777, 3780-3784, 3786-3796, 3798-3799, 7411-7412, 743, 7430, 7433-7436, 870-871, 915, 918, 921, 930, 940, 950	H04001-H04003, H04009, H04011-H04013, H04019, H04021-H04023, H04029, H04031-H04033, H04039, H04301-H04303, H04309, H04331-H04333, H04339, H04321-H04323, H04329, H04311-H04313, H04319, H04421-H04423, H04429, H04411-H04413, H04419, H04431-H04433, H04439, H0500-H05013, H05019, H05031-H05033, H05039, H05021-H05023, H05029, H05041-H05043, H05049, H0510, H05111-H05113, H05119, H05121-H05123, H05129, H040, H0400-H0403, H043, H0430-H0433, H044, H0441-H0443, H050, H0501-H0504, H051, H0511, H0512
Infectious diseases	1730-1736, 3281, 3681, 5320-22, 5239, 5440-5449, 5571, 760, 761, 769, 770-778, 7798, 7799, 903, 9250-52, 9483, 9840-9843, 9849, 11502, 11512, 11592, 1253, 1301, 1302, 13621, 1391, 37105	A1850-A1852, A1859, A3686, A3982, B0239, B0233, B0232, B0050, B0059, B0052, B0051, B0581, A711, A719, A740, B300-B303, B308, A7489, B309, A5031, A5143, A5219, A5431, A5432, A5439, A5433, B394, B395, B399, B7300, B7301, B7302, B7309, B731, B5809, B5801, A1853, B940, A5030, A5032, A5039, A5044, A5430, A710, B0053, B0181, B0230, B0234, B6013, B691, A1854, B6012
Endophthalmitis	36000-36004, 36011-36014, 36019	H44001-H44003, H44009, H44011-H44013, H44019, H44021-H44023, H44029, H44131-H44133, H44139, H44111-H44113, H44119, H21331-H21333, H21339, H33121-H33123, H33129, H44121-H44123, H44129, H16241-H16243, H16249, H4419, H1624, H3312, H440, H4400-H4402, H441, H4411-H4413
Eyelid disorders	37300-37302, 37311-37313, 3732, 37331-37334, 3734-3736, 3738-3739	H01001-H01009, H01011-H01016, H01019, H01021-H01026, H01029 H00011-H00016, H00019, H01021-H01029, H00011-H00016, H00019, H00021-H00026, H00029, H00031-H00036, H00039, H0011-H0016, H0019, H01131-H01136, H01139, H01111-H01116, H01119, H01141-H01146, H01149, H01121-H01126, H01129, H018-H019, H00, H000, H0001-H0003, H001-H010, H0100-H0102, H0111-H0114

The role of inflammation is increasingly being recognized in diseases such as atherosclerosis or Alzheimer's disease.^8^^,^^9^ Similarly, some instances of cataract or glaucoma can be caused by inflammation. Our analysis in this study is limited to entities like conjunctivitis or keratitis in which immune or infectious causes are generally overt and commonly recognized.

Medicare beneficiaries are classified as either male or female; only a few beneficiaries have missing gender data and all missing cases are suppressed. Race includes Asian, Black or African American (non-Hispanic), White (non-Hispanic), Hispanic (any race), North American Native, and other (including multiple or missing race). Few beneficiaries had missing race/ethnicity data and were excluded because their results would have been suppressed.

Racial and gender differences in use are investigated by stratifying data by race and gender individually and combining race and gender for the inflammatory and infectious eye disease category and for the individual disease subgroups for 2014 to 2018. The results are presented as percentage and 95% confidence intervals. Confidence intervals are calculated using the Clopper–Pearson (exact) method based on a binomial distribution. Denominator for percentage calculation is the total number of beneficiaries enrolled in Medicare for that calendar year. All analyses were conducted using SAS (SAS Institute, Cary, NC).

## Results

**Figure 1.**
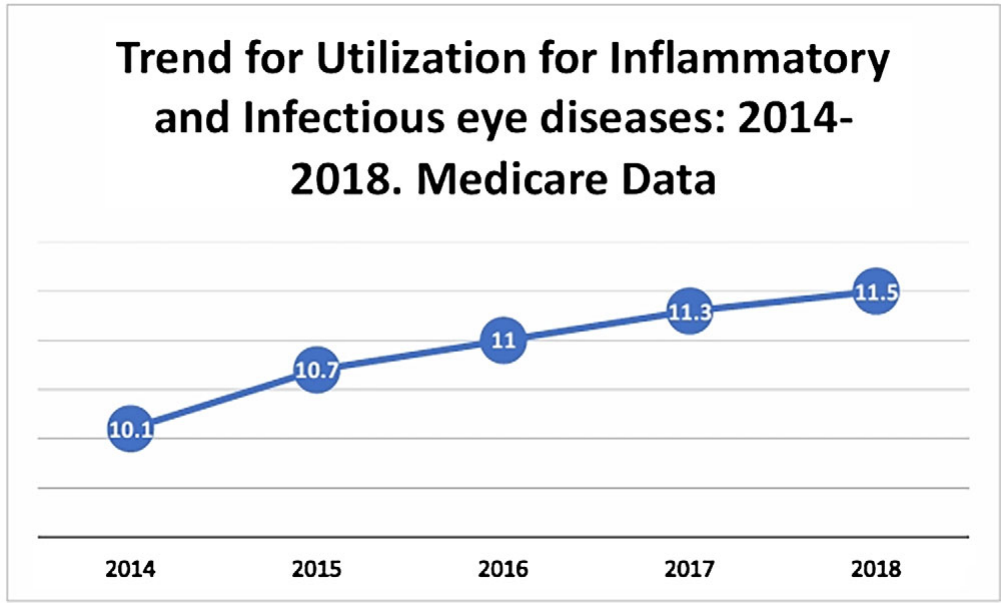
Trend for use for inflammatory and infection eye disease using Medicare data from 2014 to 2018.

There were 29 to 30 million Medicare beneficiaries enrolled for each year from 2014 to 2018. Table 2 shows the distribution of study population from 2014 to 2018. Use for inflammatory and infectious eye diseases for the total population is shown in Table 3. When usewas stratified by gender, females have higher use than males from 2014 to 2018 (Table 4). On stratification by race, African Americans have lower use, Asians have highest use, followed by Hispanics as compared with Whites from 2014 to 2018 (Table 5). When stratifying by gender and race, females have higher use for the inflammatory and infectious eye diseases than males, across all races from 2014 to 2018. Asian females have higher use than Asian males, from 2014 to 2018 (Table 6). Trend of use for inflammatory and infectious eye disease category have increased during the study period (Fig. 1). In 2014, use was 10.1%, which increased to 11.5% in 2018.

**Table 2.** Distribution of Study Population: Medicare, 2014–2018

	2014	2015	2016	2017	2018
Total	29, 974, 000	30, 028, 300	30, 424, 200	30, 239, 200	29, 909, 000
Gender					
Male	13, 358, 800	13, 402, 300	13, 584, 100	13, 526, 400	13, 395, 000
Female	16, 615, 200	16, 626, 000	16, 840, 100	16, 712, 800	16, 514, 000
Race					
White	24, 253, 300	24, 323, 800	24, 592, 500	24, 438, 700	24, 234, 800
African American	2, 897, 500	2, 866, 100	2, 886, 200	2, 821, 700	2, 711, 100
Hispanic	1, 731, 400	1, 726, 800	1, 783, 500	1, 789, 000	1, 755, 400
Asian	698, 700	714, 000	755, 400	779, 500	794, 500
North American Native	165, 700	168, 800	172, 700	177, 100	177, 100
Other	227, 500	228, 900	233, 900	233, 200	236, 100
Age, years					
18–39	833, 000	806, 100	792, 700	754, 600	708, 900
40–64	4, 349, 300	4, 272, 100	4, 204, 200	4, 040, 300	3, 802, 600
65–85	20, 844, 300	21, 057, 200	21, 553, 200	21, 683, 600	21, 759, 800
>85	3, 946, 200	3, 891, 900	3, 873, 100	3, 759, 800	3, 636, 800

**Table 3.** Medicare Use for Inflammatory and Infectious Eye Diseases for Total Population: 2014–2018

Year	Total Population	Inflammatory and Infectious Eye Disease, No. % (95% CI)	Conjunctivitis, No. % (95% CI)	Endophthalmitis, No. % (95% CI)	Eyelid Infection and Inflammation, No. % (95% CI)	Infectious Diseases, No. % (95% CI)	Keratitis, No. % (95% CI)	Lacrimal and Orbital Inflammation, No. % (95% CI)	Other Inflammatory Diseases, No. % (95% CI)
2014	29, 974, 000	3, 027, 374	1, 378, 804	29, 974	1, 198, 960	119, 896	659, 428	29, 974	179, 844
		10.1	4.60	0.1	4	0.4	2.2	0.1	0.6
		(10.1–10.1)	(4.60–4.60)	(0.1–0.1)	(4–4)	(0.4–0.4)	(2.2–2.2)	(0.1–0.1)	(0.6–0.6)
2015	30, 028, 300	3, 213, 028	1, 471, 387	30, 028	1, 261, 189	120, 113	690, 650.9	30, 028	1, 801, 698
		10.7	4.9	0.1	4.2	0.4	2.3	0.1	0.6
		(10.6–10.7)	(4.9–4.9)	(0.1–0.1)	(4.2–4.2)	(0.4–0.4)	(2.3–2.4)	(0.1–0.1)	(0.6–0.6)
2016	30, 424, 200	3, 346, 662	1, 612, 482.6	30, 424	1, 186, 543	121, 697	821, 453	30, 424	152, 121
		11	5.3	0.1	3.9	0.4	2.7	0.1	0.5
		(11–11)	(5.2–5.3)	(0.1–0.1)	(3.9–3.9)	(0.4–0.4)	(2.7–2.7)	(0.1–0.1)	(0.5–0.5)
2017	30, 239, 200	3, 417, 030	1, 602, 677.6	30, 239	1, 179, 329	151, 196	907, 176	30, 239	151, 196
		11.3	5.3	0.1	3.9	0.5	3	0.1	0.5
		11.3–11.3	(5.3–5.3)	(0.1–0.1)	(3.9–3.9)	(0.5–0.5)	(3–3)	(0.1–0.1)	(0.5–0.5)
2018	29, 909, 000	3, 439, 535	1, 585, 177	29, 909	1, 196, 360	149, 545	957, 088	29, 909	149, 545
		11.5	5.30	0.1	4	0.5	3.2	0.1	0.5
		(11.5–11.6)	(5.3–5.3)	(0.1–0.1)	(4–4)	(0.5–0.5)	(3.2–3.2)	(0.1–0.1)	(0.5–0.5)

% = Number of persons with inflammatory and infectious eye diseases per 100.

**Table 4.** Medicare Use for Inflammatory and Infectious Eye Diseases by Gender: 2014–2018

	N	Inflammatory and Infectious Eye Disease,^a^ % (95% CI)	Conjunctivitis, % (95% CI)	Endophthalmitis, % (95% CI)	Eyelid Infection and Inflammation, % (95% CI)	Infectious Diseases, % (95% CI)	Keratitis, % (95% CI)	Lacrimal and Orbital Inflammation, % (95% CI)	Other Inflammatory Diseases, % (95% CI)
2014									
Male	13, 358, 800	8.4 (8.3–8.4)	3.8 (3.8–3.8)	0.1 (0.1–0.1)	3.4 (3.4–3.5)	0.3 (0.3–0.3)	1.5 (1.5–1.5)	0.1 (0.1–0.1)	0.5 (0.5–0.5)
Female	16, 615, 200	11.5 (11.5–11.5)	5.2 (5.2–5.3)	0.1 (0.1–0.1)	4.4 (4.4–4.4)	0.4 (0.4–0.4)	2.8 (2.70–2.8)	0.2 (0.2–0.2)	0.6 (0.6–0.6)
2015									
Male	13, 402, 300	8.8 (8.8–8.8)	4.1 (4.1–4.1)	0.1 (0.1–0.1)	3.6 (3.6–3.6)	0.4 (0.4–0.4)	1.6 (1.6–1.6)	0.1 (0.1–0.1)	0.5 (0.5–0.5)
Female	16, 626, 000	12.1 (12.1–12.1)	5.5 (5.5–5.5)	0.1 (0.1–0.1)	4.6 (4.6–4.6)	0.4 (0.4–0.5)	3 (3–3)	0.2 (0.2–0.2)	0.6 (0.6–0.6)
2016									
Male	13, 584, 100	9.1 (9.1–9.2)	4.5 (4.5–4.5)	0.1 (0.1–0.1)	3.3 (3.3–3.3)	0.4 (0.4–0.4)	1.8 (1.8–1.8)	0.1 (0.1–0.1)	0.4 (0.4–0.4)
Female	16, 840, 100	12.5 (12.5–12.6)	5.8 (5.8–5.9)	0.1 (0.1–0.1)	4.3 (4.3–4.3)	0.5 (0.5–0.5)	3.4 (3.4–3.4)	0.1 (0.1–0.1)	0.6 (0.6–0.6)
2017									
Male	13, 526, 400	9.2 (9.20–9.2)	4.5 (4.5–4.6)	0.1 (0.1–0.1)	3.3 (3.3–3.3)	0.4 (0.4–0.4)	2 (2–2)	0.1 (0.1–0.1)	0.4 (0.4–0.4)
Female	16, 712, 800	13 (13–13)	5.9 (5.9–5.9)	0.1 (0.1–0.1)	4.4 (4.4–4.4)	0.5 (0.5–0.5)	3.9 (3.9–3.9)	0.1 (0.1–0.1)	0.6 (0.6–0.6)
2018									
Male	13, 395, 000	9.3 (9.30–9.4)	4.6 (4.5–4.6)	0.1 (0.1–0.1)	3.3 (3.3–3.3)	0.4 (0.4–0.4)	2.1 (2.1–2.1)	0.1 (0.1–0.1)	0.4 (0.4–0.4)
Female	16, 514, 000	13.3 (13.3–13.3)	5.9 (5.9–5.9)	0.1 (0.1–0.1)	4.5 (4.5–4.5)	0.5 (0.5–0.5)	4.1 (4.1–4.1)	0.1 (0.1–0.1)	0.6 (0.6–0.6)

a Inflammatory and Infectious eye disease category has subgroups of conjunctivitis, endophthalmitis, eyelid infection and inflammation, infectious diseases, keratitis, lacrimal and orbital inflammation, and other inflammatory diseases.

% = Number of persons with inflammatory and infectious eye diseases per 100.

**Table 5.** Medicare Use for Inflammatory and Infectious Eye Disease Category and Subgroups by Race: 2014–2018

		Inflammatory and Infectious Eye Disease,^a^ % (95% CI)	Conjunctivitis, % (95% CI)	Eyelid Infection and Inflammation, % (95% CI)	Keratitis, % (95% CI)	Infectious Diseases, % (95% CI)	Lacrimal and Orbital Inflammation, % (95% CI)	Endophthalmitis, % (95% CI)	Other,^b^ % (95% CI)
2014									
White	24, 253, 300	10.2 (10.20–10.20)	4.4 (4.4–4.4)	4.2 (4.2–4.2)	2.2 (2.2–2.2)	0.4 (0.4–0.4)	0.1 (0.1–0.1)	0.1 (0.1–0.1)	0.5 (0.5–0.5)
Black	2, 897, 500	7.9 (7.9–7.9)	4.2 (4.1–4.2)	2.2 (2.2–2.2)	1.3 (1.3–1.3)	0.2 (0.2–0.2)	0.2 (0.2–0.2)	0.2 (0.2–0.2)	1 (1–1)
Hispanic	1, 731, 400	10.8 (10.7–10.8)	6.1 (6.1–6.1)	3.3 (3.3–3.3)	2.3 (2.3–2.3)	0.3 (0–2–0.3)	0.2 (0.2–0.2)	0.1 (0.1–0.1)	0.6 (0.6–0.6)
Asian	698, 700	15 (14.9–15.1)	8.8 (8.7–8.8)	4.7 (4.6–4.7)	3.8 (3.7–3.8)	0.3 (0–3–0.4)	0.2 (0.2–0.2)	0.1 (0.1–0.1)	0.8 (0.8–0.8)
NAN	165, 700	9.5 (9.40–9.7)	5.6 (5.5–5.6)	2.5 (2.4–2.6)	1.8 (1.7–1.9)	0.2 (0.2–0.2)	0.2 (0.2–0.2)	0.1 (0.1–0.1)	0.7 (0.6–0.7)
Other	227, 500	10.9 (10.8–11)	5.5 (5.4–5.6)	3.9 (3.9–4)	2.4 (2.3–2.5)	0.3 (0.3–0.4)	0.2 (0.2–0.2)	0.1 (0.0–0.1)	0.7 (0.6–0.7)
2015									
White	24, 323, 800	10.7 (10.7–10.8)	4.7 (4.7–4.7)	4.4 (4.4–4.4)	2.4 (2.4–2.4)	0.4 (0.4–0.4)	0.1 (0.1–0.1)	0.1 (0.0–0.1)	0.5 (0.5–0.5)
Black	2, 866, 100	8.4 (8.3–8.4)	4.5 (4.5–4.5)	2.3 (2.2–2.3)	1.5 (1.5–1.5)	0.3 (0.3–0.3)	0.2 (0.2–0.2)	0.1 (0.1–0.1)	1 (1–1)
Hispanic	1, 726, 800	11.2 (11.2–11.3)	6.4 (6.4.–6.5)	3.5 (3.4–3.5)	2.3 (2.4–2.4)	0.3 (0.3–0.3)	0.2 (0.2–0.2)	0.1 (0.1–0.1)	0.6 (0.6–0.6)
Asian	714, 000	15.3 (15.2–15.3)	8.8 (8.7–8.8)	4.7 (4.8–4.9)	3.9 (3.9–3.9)	0.4 (0.3–0.4)	0.2 (0.2–0.2)	0.1 (0.1–0.1)	0.8 (0.8–0.8)
NAN	168, 800	9.9 (9.8–10.1)	5.9 (5.8–6)	2.6 (2.5–2.6)	1.8 (1.8–1.9)	0.3 (0.3–0.3)	0.2 (0.1–0.2)	0.1 (0.1–0.1)	0.7 (0.6–0.7)
Other	228, 900	11.5 (11.4–11.7)	5.9 (5.8–6)	4.2 (4.1–4.3)	2.6 (2.5–2.7)	0.4 (0.3–0.4)	0.2 (0.1–0.2)	0.1 (0.1–0.1)	0.7 (0.6–0.7)
2016									
White	24, 592, 500	11.1 (11.1–11.1)	5 (5–5)	4.1 (4.1–4.1)	2.8 (2.8–2.8)	0.5 (0.5–0.5)	0.1 (0.1–0.1)	0.1 (0.0–0.1)	0.5 (0.5–0.5)
Black	2, 886, 200	8.7 (8.7–8.8)	4.9 (4.9–4.9)	2.1 (2.1–2.1)	1.7 (1.6–1.7)	0.3 (0.3–0.3)	0.1 (0.1–0.1)	0.1 (0.0–0.1)	1 (1–1)
Hispanic	1, 783, 500	11.5 (11.5–11.6)	6.9 (6.8.–6.9)	3.2 (3.2–3.2)	2.6 (2.6–2.6)	0.3 (0.3–0.3)	0.1 (0.1–0.1)	0.1 (0.0–0.1)	0.5 (0.5–0.5)
Asian	755, 400	15.3 (15.2–15.4)	9.2 (9.1–9.3)	4.3 (4.3–4.4)	3.8 (3.8–3.9)	0.4 (0.4–0.4)	0.2 (0.1–0.2)	0.1 (0.0–0.1)	0.7 (0.6–0.7)
NAN	172, 700	10.1 (9.9–10.2)	6.2 (6.1.–6.3)	2.2 (2.1–2.3)	2.1 (2–2.1)	0.3 (0.3–0.3)	0.1 (0.1–0.1)	0.1 (0.1–0.1)	0.6 (0.6–0.6)
Other	233, 900	12.1 (12–12.2)	6.5 (6.4–6.6)	3.9 (3.8–4)	2.8 (2.8–2.9)	0.4 (0.4–0.4)	0.1 (0.1–0.1)	0.1 (0.1–0.1)	0.6 (0.6–0.6)
2017									
White	24, 438, 700	11.4 (11.4–11.5)	5.1 (5.1–5.1)	4.1 (4.1–4.2)	3.1 (3.1–3.1)	0.5 (0.5–0.5)	0.1 (0.1–0.1)	0.1 (0.1–0.1)	0.5 (0.5–0.5)
Black	2, 821, 700	8.9 (8.9–9)	4.9 (4.9–4.9)	2 (2–2)	2 (1.9–2)	0.3 (0.3–0.3)	0.1 (0.1–0.1)	0.1 (0.1–0.1)	1 (1–1)
Hispanic	1, 789, 000	11.6 (11.6–11.6)	6.8 (6.8.–6.8)	3.1 (3.1–3.2)	2.9 (2.9–2.9)	0.3 (0.3–0.3)	0.1 (0.1–0.1)	0.1 (0.0–0.1)	0.5 (0.5–0.5)
Asian	779, 500	15.6 (15.5–15.5)	9.2 (9.2–9.3)	4.2 (4.2–4.3)	4.2 (4.2–4.3)	0.4 (0.4–0.4)	0.1 (0.1–0.1)	0.1 (0.0–0.1)	0.7 (0.7–0.7)
NAN	177, 100	10.1 (10.1–10.4)	6.2 (6.1.–6.3)	2.1 (2.1–2.2)	2.3 (2.2–2.4)	0.3 (0.3–0.4)	0.1 (0.1–0.1)	0.1 (0.1–0.1)	0.6 (0.6–0.7)
Other	233, 200	12.6 (12.5–12.7)	6.6 (6.5–6.7)	3.9 (3.8–4)	3.3 (3.2–3.4)	0.4 (0.4–0.4)	0.1 (0.1–0.1)	0.1 (0.1–0.1)	0.6 (0.6–0.6)
2018									
White	24, 234, 800	11.7 (11.7–11.7)	5.1 (5.1–5.1)	4.3 (4.3–4.3)	3.3 (3.3–3.3)	0.5 (0.5–0.5)	0.1 (0.1–0.1)	0.1 (0.1–0.1)	0.5 (0.5–0.5)
Black	2, 711, 100	9 (9–9)	4.8 (4.8–4.9)	2.1 (2.1–2.1)	2.1 (2–2.1)	0.3 (0.3–0.3)	0.1 (0.1–0.1)	0.1 (0.1–0.1)	1 (1–1)
Hispanic	1, 755, 400	11.8 (11.7–11.8)	6.8 (6.8.–6.8)	3.2 (3.2–3.2)	3.1 (3–3.1)	0.3 (0.3–0.3)	0.1 (0.1–0.1)	0.1 (0.0–0.1)	0.5 (0.5–0.5)
Asian	794, 500	15.6 (15.4–15.6)	9 (8.9–9.1)	4.2 (4.1–4.2)	4.4 (4.4–4.4)	0.4 (0.4–0.4)	0.4 (0.4–0.4)	0.1 (0.0–0.1)	0.7 (0.7–0.7)
NAN	177, 100	10.2 (10.3–10.5)	6.3 (6.2–6.4)	2.1 (2.1–2.2)	2.4 (2.3–2.4)	0.3 (0.3–0.4)	0.1 (0.1–0.1)	0.1 (0.1–0.1)	0.6 (0.6–0.7)
Other	236, 100	12.9 (12.8–13.1)	6.7 (6.6–6.8)	4.1 (4–4.2)	3.5 (3.5–3.6)	0.4 (0.4–0.4)	0.1 (0.1–0.1)	0.1 (0.1–0.1)	0.6 (0.6–0.7)

NAN, North American Native.

a Inflammatory and Infectious eye disease category has subgroups of conjunctivitis, endophthalmitis, eyelid infection and inflammation, infectious diseases, keratitis, lacrimal and orbital inflammation, and other inflammatory diseases.

b Other = other inflammatory diseases.

% = Number of persons with inflammatory and infectious eye diseases per 100.

**Table 6.** Medicare Use for Inflammatory and Infectious Eye Disease Category by Race and Gender: 2014–2018

	2014		2015		2016		2017		2018	
	No.	% (95% CI)	No.	% (95% CI)	No.	% (95% CI)	No.	% (95% CI)	No.	% (95% CI)
All races										
Male	13, 358, 800	8.4 (8.3–8.4)	13, 402, 300	8.8 (8.8–8.8)	13, 584, 100	9.1 (9–9.1)	13, 526, 400	9.2 (9.2–9.2)	13, 395, 000	9.4 (9.3–9.4)
Female	16, 615, 200	11.5 (1.5–11.5)	16, 626, 000	12.1 (12.1–12.1)	16, 840, 100	12.5 (12.5–12.6)	16, 712, 800	13 (13–13)	16, 514, 000	13.3 (13.3–13.3)
Asian										
Male	297, 800	13 (12.9–13.1)	304, 300	13.2 (13.1–13.3)	321, 200	13.3 (13.2–13.5)	329, 800	13.5 (13.4–13.6)	334, 000	13.4 (13.3–13.5)
Female	400, 900	16.6 (16.4–16.6)	409, 700	16.8 (16.7–16.9)	434, 300	16.8 (16.7–16.9)	449, 700	17.2 (17–17.3)	460, 400	17 (16.9–17.1)
Black										
Male	1, 269, 300	5.8 (5.7–5.8)	1, 258, 900	6.1 (6.1–6.2)	1, 267, 400	6.5 (6.4–6.5)	1, 245, 900	6.5 (6.5–6.6)	1, 200, 600	6.6 (6.5–6.6)
Female	1, 628, 200	9.6 (9.5–9.6)	1, 607, 200	10.1 (10.1–10.2)	1, 618, 800	10.5 (10.5–10.6)	1, 575, 800	10.8 (10.8–10.9)	1, 510, 500	10.9 (10.9–11)
Hispanic										
Male	805, 800	8.7 (8.6–8.7)	806, 600	9 (8.9–9.1)	831, 300	9.3 (9.3–9.4)	834, 000	9.3 (9.3–9.4)	818, 000	9.3 (9.3–9.4)
Female	925, 600	12.6 (12.6–12.7)	920, 100	13.2 (13.1–13.2)	952, 200	13.4 (13.3–13.5)	955, 000	13.6 (13.5–13.6)	937, 400	13.9 (13.8–13.9)
NAN										
Male	73, 500	7.7 (7.5–7.9)	74, 700	8.1 (7.9–8.3)	76, 300	8.1 (7.9–8.3)	78, 200	8.2 (8–8.4)	78, 000	8.3 (8.1–8.5)
Female	92, 200	11 (10.8–11.2)	94, 100	11.4 (11.2–11.6)	96, 400	11.6 (11.4–11.8)	99, 000	11.8 (11.6–12)	99, 100	11.9 (11.7–12.1)
White										
Male	10, 801, 200	8.5 (8.5–8.5)	10, 847, 000	9 (9–9)	10, 976, 000	9.2 (9.2–9.2)	10, 927, 800	9.4 (9.4–9.4)	10, 852, 300	9.5 (9.5–9.6)
Female	13, 452, 000	11.5 (11.5–11.5)	13, 476, 800	12.2 (12.1–12.2)	13, 616, 400	12.6 (12.6–12.6)	13, 510, 900	13.1 (13.1–13.1)	13, 382, 500	13.4 (13.4–13.4)
Other										
Male	111, 300	9 (8.8–9.2)	110, 900	9.7 (9.5–9.9)	111, 900	10.1 (9.9–10.2)	110, 800	10.4 (10.3–10.6)	112, 000	10.8 (10.6–10.9)
Female	116, 200	12.7 (12.5–12.9)	118, 100	13.3 (13.1–13.5)	122, 000	13.9 (13.7–14.1)	122, 400	14.6 (14.4–14.8)	124, 000	14.9 (14.7–15.1)

NAN, North American Native.

% = Number of persons with inflammatory and infectious eye diseases per 100.

## Discussion

Our study aimed to examine the effects of race and gender on Medicare use for inflammatory and infectious eye diseases using Medicare data from the VEHSS. The findings of this study revealed race and gender disparities in health care use among Medicare beneficiaries with these ocular conditions. These differences may be due either to differences in use or differences in prevalence. Because we have used Medicare, an administrative database, we have reported our results as use.

Race was found to be a significant factor influencing Medicare use patterns for inflammatory and infectious eye diseases. Our study observed that African American beneficiaries had lower use as compared with use by Whites. Asians and Hispanics have higher use.

Eye care use in patients with diabetes using Medicare data has shown that African Americans have lower use, which is similar to our study findings of African Americans having lower use for inflammatory and infectious eye diseases.^10^ African Americans are less likely to visit an eye doctor than White patients.^11^ Behavioral differences may influence use of health care in African Americans.^7^ These include the fear of loss of income when seeing an eye care provider, lack of transportation, and barriers related to acceptability, fear of treatment with dignity, and concerns regarding discrimination.^6^ Lack of awareness about vision, eye health conditions, and availability of eye care services may also lead to less use of eye health providers among African Americans.^12^^,^^13^ African Americans have higher prevalence of ocular inflammatory and infectious diseases^14^^,^^15^; hence, lower Medicare use represents a barrier to access eye care and not lower disease.

Previous studies have found Hispanics have lower use than Whites,^16^^,^^17^ whereas we have found higher use for Hispanics. The reason for these differences may be that our study has examined inflammatory and infectious eye diseases, whereas previous studies have been focused on general eye care, use of eye glasses, and eye care in patients with diabetes.^16^^,^^17^

Studies regarding use for eye care in Asians have yielded mixed results. Studies that have compared Asians as the main racial group with Whites have shown higher use in Asians as compared with Whites, similar to our study results,^18^ although other studies have shown lower use.^16^^,^^17^ The reason for this may be that most studies do not have Asians as a major racial group and, therefore, may lack the power to detect a difference. Our study has a larger number of Asians (average number of 748, 000 as compared with 161 for the study by Varadaraj et al.^16^ and 1462 for the study by Canedo et al.^17^) and, therefore, was able to detect these differences.

Enrollment in a health insurance program like Medicare depends on the patient's ability to understand and obtain health insurance, known as health insurance literacy. Heath insurance literacy helps patients to choose an insurance program that aligns with their needs and preferences and provides them with lower costs and better coordinated care.^19^ Health literacy is especially low for racial minorities as compared with Whites. This low health literacy combined with the complexity of health insurance programs results in a barrier to health care for racial minorities.^20^ Medicare has the traditional Medicare program, which is provided by the federal government, and a Medicare Advantage program, which is private. Patients with high health literacy may choose Medicare Advantage or traditional Medicare, whichever is suitable for their health care needs and provides them with lower cost and better coverage. Owing to their low health insurance literacy, racial minorities may enroll in traditional Medicare because many times they are not aware of Medicare Advantage plans.^19^ Traditional Medicare may not be their best fit in the providing care that best suits their needs, and this lack may result in racial minorities not using Medicare-provided health care, even when needed, resulting in racial disparities.

Gender disparities were also evident in Medicare use for inflammatory and infectious eye diseases. Female beneficiaries demonstrated higher use rates as compared with males. Higher use by females may be due to their greater awareness of eye symptoms, a lower threshold of symptoms before seeking care, and higher comfort level in seeking eye care.^21^^,^^22^

When comparing individual inflammatory and infectious eye disease conditions by gender with the published literature, there are differences in number of cases for males and females. Women have greater keratitis,^23^^,^^24^ scleritis,^25^^–^^27^ episcleritis,^26^^,^^27^ uveitis,^2^^,^^28^^,^^29^ orbital inflammation,^30^^,^^31^ and blepharitis.^32^

In one study, males had a higher number of keratitis cases.^33^ The difference in that study result from our study could be due to the study population. That study included people with commercial insurance and focused on individuals with fungal keratitis, whereas our study population is from Medicare, which is government-funded insurance. Our study also includes patients with any diagnosis of keratitis, not just fungal keratitis.

Another study reported that the number of endophthalmitis^34^ cases was lower in females, whereas our study did not find a difference in number of cases between males and females. This discrepancy may be because that study examined endophthalmitis cases after cataract surgery, whereas our study examines all endophthalmitis cases.

When comparing individual inflammatory and infectious eye disease conditions for race, similar to our study, Whites reportedly had a higher number of episcleritis^25^ or uveitis cases.^29^ Unlike our study, Blacks had a higher number of cases of endophthalmitis^34^^,^^35^ as compared with Whites. These differences may be because both these studies included endophthalmitis cases after cataract surgery and were done from 1991 to 2004^35^ and in 2003 and 2004.^34^ In contrast, our study included endophthalmitis cases regardless of beneficiary undergoing prior cataract surgery and is based on data from 2014 to 2018. There were a smaller number of uveitis cases in Hispanics,^28^ which differs from our study findings, where we find higher number of uveitis cases as compared with Whites. However, the number of uveitis cases in this study was only 4. Thus, differences reported in the study results may be due to by chance alone.

Trend for use for infectious and inflammatory eye diseases has increased in our study from 2014 to 2018. This finding is similar to previous studies, which have reported an increase in inflammatory eye diseases.^3^^,^^28^^,^^35^ The reason for this increase may be an increase in the percentage of individuals greater than 65 years of age over the study period.^36^ There is also a higher burden of autoimmune eye disease in this age group, ^2^ resulting in an increase in the number of cases. An increase in the overall diagnosis of autoimmune diseases^37^ may also result in an increase in the number of cases. Another reason could be the change from ICD-9 to ICD-10 codes in 2015. The ICD-10 has more codes for inflammatory and infectious eye diseases as compared with the ICD-9. This change might increase the number of cases being identified as inflammatory and infectious eye diseases from 2014 to 2018.

The primary strength of our study is the use of the Medicare database. Medicare is an excellent database to study disparities because it is national and includes large numbers of beneficiaries. There are sufficient numbers of racial minorities to identify differences in use, which would have been missed when using smaller databases.^38^ The majority of previous studies have examined differences between African Americans and Whites, or Hispanic and Whites; less is known for Asians and North American Natives.^39^ We have identified and shown how the use varies for Asians and North American Natives. Medicare categories for race and ethnicity are valid when compared with self-reported race/ethnicity.^40^

The limitation of our study is using an administrative database. The identification of inflammatory and infectious eye disease relies on ICD-9 and -10 codes. ICD-9 and -10 codes are not always accurate and we do not have clinical data to validate the diagnoses; however, we do not believe that this inaccuracy should disproportionately affect one sex or racial group to the point that it would introduce bias in the study results. Medicare's race and ethnicity data are less accurate for American Indian/Alaska Native, Asian/Pacific Islander, or Hispanic participants. This factor may limit the ability to assess health disparities.^23^ A second race variable was added to Medicare at the Research Triangle Institute to improve classification of Hispanics and Asians/Pacific Islanders. The Research Triangle Institute race variable has been shown to be accurate for identifying Hispanics, non-Hispanic Whites, or Blacks for both males and females.^25^

One of the factors that contributes to inaccuracy is missing information for the study population, and missing data have been excluded from our study. Hence, our study results would still be accurate and provide useful information at a national level. With the high enrollment in Medicare (close to 90%), our study population is representative of individuals 65 years of age and older. Uveitis is considered by some to be the prototypic ocular inflammatory disease. It is captured in this database within the subgroup of other inflammatory conditions, with the exceptions of panuveitis and sympathetic uveitis, which are contained within endophthalmitis.

## Conclusions

Our study highlights race and gender disparities in Medicare use for inflammatory and infectious eye diseases. Further research is needed to examine the impact of these disparities on clinical outcomes for individuals affected by inflammatory and infectious eye diseases. Our findings stress the need for targeted interventions to promote equitable access to eye car.
